# Supplementary material for: Cortical beta coherence provides a stronger non-invasive predictor of movement vigor than local beta power
Source: J Neuroeng Rehabil. 2026 Apr 11;23:169. doi: 10.1186/s12984-026-01985-w (PMC13214113; doi:10.1186/s12984-026-01985-w)
Supplement: Supplementary file 2 — Supplementary Material 2. [file 12984_2026_1985_MOESM2_ESM.docx]

1. EEG preprocessing

First, data was downsampled to 256 Hz. Then, a 1-49 Hz bandpass, two-pass, finite impulse response (FIR) filter was applied. Noisy channels were automatically detected using the Clean raw data algorithm from EEGLAB with the following parameters: signal flat for more than 4 s, standard deviation of high-frequency noise superior to 4, and correlation with nearby channels inferior to 0.85. The original signal from those channels was removed and interpolated based on the activity from their neighboring electrodes. Data was then segmented into epochs of 14 s duration locked around go cue onset (-11.5 s to +2.5 s). This period included the time window of NF presentation and movement preparation and execution, with an additional minimum 1.5 s period before NF presentation, which was used as baseline period for subsequent analyses. Independent component analysis (ICA) was run on all epochs of all conditions, for each participant separately, using runica algorithm from EEGLAB (with pca parameter set to 60). Artefactual components related to eye movements were identified based on their anterior location, spurious occurrences and low frequency dominant spectrum [41] and removed for each participant and session (min number of components removed = 0, max = 2). The signal was then re-referenced to the average of all EEG electrodes, at each time point.

1. Computation of β power in the sensor space

Time-frequency decomposition was conducted using .compute_tfr() function with the following parameters: method=”morlet”, freqs=[15:25] with 1Hz step, n_cycles=[15:25]/2, average=False, decim=2, output=”complex”. “Freqs” parameter was modified to [8:15] to extract low β power, and to [25:35 Hz] to extract high β power. For each condition (Fast/Slow and β sub-band (standard, low, high), we obtained a 140 (trials) x 128 (channels) x 7 (time windows) β power matrix.

1. Computation of β iCoh and β power in the source space

Source estimates were obtained per epoch with the apply_inverse_epochs function using a pre-computed inverse operator based on the fsaverage template head model (boundary-element forward model and oct6 source space), a noise covariance matrix computed from −2 to 0 s before NF onset regularized via a shrinkage estimator (method='shrunk'), minimum norm estimate (MNE) as the inverse method, fixed normal orientations (pick_ori='normal'), and lambda2=1 (SNR = 1); depth weighting was set to 3. Normal orientation was chosen to limit inter-individual variability that could be induced with free orientations and affect between-subject comparisons of β activity in Fast and Slow conditions.
